# Patterned functional network disruption in amyotrophic lateral sclerosis

**DOI:** 10.1002/hbm.24740

**Published:** 2019-07-26

**Authors:** Stefan Dukic, Roisin McMackin, Teresa Buxo, Antonio Fasano, Rangariroyashe Chipika, Marta Pinto‐Grau, Emmet Costello, Christina Schuster, Michaela Hammond, Mark Heverin, Amina Coffey, Michael Broderick, Parameswaran M. Iyer, Kieran Mohr, Brighid Gavin, Niall Pender, Peter Bede, Muthuraman Muthuraman, Edmund C. Lalor, Orla Hardiman, Bahman Nasseroleslami

**Affiliations:** ^1^ Academic Unit of Neurology, Trinity Biomedical Sciences Institute, Trinity College Dublin University of Dublin Dublin Ireland; ^2^ Department of Neurology University Medical Centre Utrecht Brain Centre, Utrecht University Utrecht The Netherlands; ^3^ Computational Neuroimaging Group, Trinity Biomedical Sciences Institute, Trinity College Dublin University of Dublin Dublin Ireland; ^4^ Trinity Centre for Bioengineering, Trinity College Dublin University of Dublin Dublin Ireland; ^5^ Movement disorders and Neurostimulation, Biomedical Statistics and Multimodal Signal Processing Unit, Department of Neurology Johannes‐Gutenberg‐University Hospital Mainz Germany; ^6^ Trinity College Institute of Neuroscience, Trinity College Dublin University of Dublin Dublin Ireland; ^7^ Department of Biomedical Engineering and Department of Neuroscience University of Rochester Rochester New York; ^8^ Department of Neurology Beaumont Hospital Dublin Ireland

**Keywords:** amyotrophic lateral sclerosis, EEG, functional connectivity, motor neurone disease, resting state, source localisation

## Abstract

Amyotrophic lateral sclerosis (ALS) is a progressive neurodegenerative disease primarily affecting motor function, with additional evidence of extensive nonmotor involvement. Despite increasing recognition of the disease as a multisystem network disorder characterised by impaired connectivity, the precise neuroelectric characteristics of impaired cortical communication remain to be fully elucidated. Here, we characterise changes in functional connectivity using beamformer source analysis on resting‐state electroencephalography recordings from 74 ALS patients and 47 age‐matched healthy controls. Spatiospectral characteristics of network changes in the ALS patient group were quantified by spectral power, amplitude envelope correlation (co‐modulation) and imaginary coherence (synchrony). We show patterns of decreased spectral power in the occipital and temporal (δ‐ to β‐band), lateral/orbitofrontal (δ‐ to θ‐band) and sensorimotor (β‐band) regions of the brain in patients with ALS. Furthermore, we show increased co‐modulation of neural oscillations in the central and posterior (δ‐, θ‐ and γ_l_‐band) and frontal (δ‐ and γ_l_‐band) regions, as well as decreased synchrony in the temporal and frontal (δ‐ to β‐band) and sensorimotor (β‐band) regions. Factorisation of these complex connectivity patterns reveals a distinct disruption of both motor and nonmotor networks. The observed changes in connectivity correlated with structural MRI changes, functional motor scores and cognitive scores. Characteristic patterned changes of cortical function in ALS signify widespread disease‐associated network disruption, pointing to extensive dysfunction of both motor and cognitive networks. These statistically robust findings, that correlate with clinical scores, provide a strong rationale for further development as biomarkers of network disruption for future clinical trials.

**Abbreviations**
(a) FDR: (Adaptive) false discovery rate
(f)MRI: (Functional) magnetic resonance imaging
AEC: Amplitude envelope correlation
ALS: Amyotrophic lateral sclerosis
ALSFRS‐R: ALS Functional rating scale‐revised
AUC(ROC): Area under the curve (of the receiver operating characteristics curve)
EBI: Empirical Bayesian inference
EEG: Electroencephalography
iCoh: Imaginary coherence
M: Module
NMF: Non‐negative matrix factorisation
ROI: Region of interest
TMS: Transcranial magnetic stimulation

## 1 INTRODUCTION

Amyotrophic lateral sclerosis (ALS) is a neurodegenerative disease of upper and lower motor neurons resulting in progressive loss of bulbar and limb function (Hardiman, Van Den Berg, & Kiernan, 2011). Although originally considered a disease exclusively of the motor system (Achi & Rudnicki, 2012), widespread nonmotor (Bede et al., 2013) and subcortical (Bede et al., 2018) structural changes are now recognised (Douaud, Filippini, Knight, Talbot, & Turner, 2011). Clinical and neuroimaging evidence confirms extensive involvement of motor (Douaud et al., 2011; Nasseroleslami et al., 2019; Proudfoot, van Ede, et al., 2018) and cognitive (Iyer et al., 2015; McMackin, Dukic, et al., 2019) pathways and networks. These network impairments manifest as measurable changes in cortical connectivity, informing altered dynamics within different networks, which may lead to widespread changes in neural signalling beyond the regions of direct disease pathology.

Functional magnetic resonance imaging (fMRI) studies have identified increased connectivity in the sensorimotor networks of ALS patients (Agosta et al., 2011; Douaud et al., 2011) based on blood oxygen‐level‐dependent signal. However, network impairment can also be interrogated using neuroelectric signals captured with electroencephalography (EEG). These signals appear in varying frequency bands and can differ substantially across networks (Siegel, Donner, & Engel, 2012). This variability is due to the complex hierarchal organisation of projections between different granular layers forming connections between different areas (Barbas, 2015; Kopell, Kramer, Malerba, & Whittington, 2010), which oscillate at either lower or higher frequencies (Jensen, Bonnefond, Marshall, & Tiesinga, 2015). These spectral signatures of connectivity require high temporal resolution and cannot be captured by fMRI (Hipp, Hawellek, Corbetta, Siegel, & Engel, 2012; Laufs, 2008).

Previous EEG studies have shown altered patterns, such as increased frontal‐to‐parietal connectivity in ALS (Blain‐Moraes, Mashour, Lee, Huggins, & Lee, 2013; Iyer et al., 2015; Nasseroleslami et al., 2019). To date, however, there have been limited attempts to localise abnormal EEG patterns to specific brain regions. A recent magnetoencephalography study in ALS has focussed on slow/broadband fMRI‐like activity, and has demonstrated widespread changes within the posterior brain regions (Proudfoot, Colclough, et al., 2018). However, because network interactions are often marked by narrow band cortical oscillations (Buzsáki & Draguhn, 2004), it has not been possible to address the spectral aspects of ALS‐specific changes in brain networks using broadband signals. Moreover, although source‐space studies that use frequency‐specific analysis have also been performed in ALS (Fraschini et al., 2018; Sorrentino et al., 2018), the phase‐ and amplitude‐based connectivity profiles of specific brain networks affected by ALS remain to be established.

The majority of the commonly used EEG connectivity measures falls into two categories: amplitude‐based and phase‐based indices. Within a given frequency‐band, these two different groups of measures reflect two conceptually different aspects of the cortical communication. Amplitude‐based measures are predominantly used to quantify co‐modulation of the oscillatory activity in distinct brain areas at infra‐slow rates (<0.1 Hz), which are shown to resemble slow co‐modulations observed in resting‐state fMRI (Brookes et al., 2011; Tagliazucchi, von Wegner, Morzelewski, Brodbeck, & Laufs, 2012). These fluctuations seem to emerge from the regulation and coordination of the network activity for an (upcoming) functionally distinct task in the brain at larger temporal and spatial scales; therefore, reflecting the functional organisation of the brain networks (Leopold, Murayama, & Logothetis, 2003; Munk, Roelfsema, König, Engel, & Singer, 1996; Siegel et al., 2012). Phase‐based coupling likely informs on facilitation and regulation of communication between distinct brain areas on faster timescales (Engel, Gerloff, Hilgetag, & Nolte, 2013; Siegel et al., 2012). In principle, these two measures are independent of one another (Bruns, Eckhorn, Jokeit, & Ebner, 2000). For instance, the activity in two brain regions can strongly co‐vary, albeit their phase values being randomly distributed. However, these two types of measures and their corresponding underlying mechanisms seem to interact and work together; with the amplitude‐based coupling indicating the priming of the activation of brain areas needed for an upcoming task, and the phase‐based coupling indicating the instantaneous synchronous influences in the networks (Engel et al., 2013). Nevertheless, exploring brain dynamics exclusively using either amplitude‐ or phase‐based connectivity measure provides limited insights into the underlying functional changes and ALS pathophysiology in general.

To date, evidence of correlation between the brain network impairments in ALS observed from neuroelectric signals and clinical scores of motor and cognitive function has been limited. In addition to this, the observed changes have not discriminated between the traditionally defined clinical ALS subgroups (e.g., bulbar‐ vs. spinal‐onset ALS or ALS with the presence or absence of the pathologic hexanucleotide expansion in the *C9ORF72* gene) (Iyer et al., 2017; Nasseroleslami et al., 2019).

Here, we have reconstructed resting‐state brain activity and performed functional connectivity analysis using both amplitude‐ and phase‐based measures in a large group of ALS patients and healthy controls. Our findings correlate with clinical measures, providing robust evidence that measurement of functional connectivity can be used as a complementary investigative tool to interrogate ALS‐associated changes in brain networks.

## 2 METHODS

### 2.1 Ethical approval

Approval was obtained from the ethics committee of Beaumont Hospital, Dublin, Ireland (REC reference: 13/102) and the Tallaght Hospital/St. James's Hospital Joint Research Ethics Committee (REC) (REC reference: 2014 Chairman's Action 7, CRFSJ 0046) for St. James's Hospital, Dublin, Ireland. The experimental procedure conformed to the Declaration of Helsinki. All participants provided written informed consent before taking part in the experiments.

### 2.2 Participants

#### 2.2.1 Patient recruitment

Patients with ALS were recruited from the National ALS clinic in Beaumont Hospital, Dublin. Healthy controls were recruited from an existing control cohort of a neuropsychology study in ALS (Burke et al., 2017).

#### 2.2.2 Inclusion criteria

All ALS patients were within the first 18 months of their diagnosis and fulfilled the revised El Escorial diagnostic criteria for possible, probable, or definite ALS (Ludolph et al., 2015).

#### 2.2.3 Exclusion criteria

Patients diagnosed with primary lateral sclerosis, progressive muscular atrophy, flail arm/leg syndromes, prior transient ischemic attacks, multiple sclerosis, stroke, epilepsy, seizure disorder, brain tumours, structural brain abnormalities, other neurodegenerative conditions and other medical morbidities, such as human immunodeficiency virus, were excluded.

#### 2.2.4 The demographic profile of patients and controls

A total of 56 ALS patients with spinal onset (m/f: 41/15; mean age: 57.9 ± 12.2 years), 15 patients with bulbar onset (m/f: 10/5; age: 59.0 ± 8.4 years) and three patients with respiratory onset (m/f: 2/1; age: 62.0 ± 5.3 years) were recruited, along with 47 healthy controls (m/f: 15/32; age: 58.4 ± 12.3) (see Table 1). Patients and controls were matched for age (Mann–Whitney *U* test, *p* = .87). The post hoc analysis of gender‐imbalance (Chi‐square test, *p* < .001) using two‐way ANOVA (analysis of variance) showed no significant interaction effects on the main findings (Figure S1, Supporting Information).

**Table 1:** Breakdown of demographics

Group	*N*	Male	Female	Age (years)^a^	Disease duration (days)^a^	EEG delay (days)^a^	ALSFRS‐R^a^
Controls	47	15^b^	32^b^	58.4 ± 12.3			
ALS							
ALL	74	53^b^	21^b^	58.3 ± 11.3	694 ± 623	221 ± 327	37.5 ± 6.5 (*n* = 61)
Spinal	56	41	15	57.9 ± 12.2	736 ± 688	239 ± 370	37.4 ± 6.2 (*n* = 47)
Bulbar	15	10	5	59.0 ± 8.4	497 ± 225	185 ± 100	38.5 ± 8.4 (*n* = 11)
Thoracic	3	2	1	62.0 ± 5.3	890 ± 617	79 ± 46	35.0 ± 5.6 (*n* = 3)
*C9ORF72*+	7	4	3	61.0 ± 7.4	908 ± 917	385 ± 408	34.8 ± 9.5 (*n* = 6)
*C9ORF72*−	66	49	17	58.4 ± 11.2	673 ± 593	204 ± 318	37.8 ± 6.3 (*n* = 54)

*Note*. Disease duration is the time interval between the estimated symptom onset and the EEG recording. EEG delay is the time interval between the date of diagnosis and the EEG recording.

Abbreviations: ALSFRS‐R, amyotrophic lateral sclerosis functional rating scale‐revised; C9ORF72± , presence/absence of the repeat expansion in the Chromosome 9 open reading frame 72; EEG, electroencephalography.

a Numbers show mean ± *standard deviation*.

b Effects of gender imbalance were found insignificant.

#### 2.2.5 Genetic profile

Seven (m/f: 4/3; age: 61.0 ± 7.4) of 73 genetically tested patients had the hexanucleotide repeat expansion in *C9ORF72* (see Table 1).

### 2.3 Experiment

#### 2.3.1 Experimental paradigm

The experiment was resting state with eyes open, divided into three 2‐min recording blocks, allowing for rest between blocks. Subjects were seated in a comfortable chair, asked to relax and let their mind wander, while they fixate their gaze at the letter X (6 × 8 cm^2^) printed on an A4 sheet of paper placed approximately 1 m in front of them.

#### 2.3.2 EEG acquisition

EEG data with 128 channels were collected using the Biosemi ActiveTwo system (Biosemi B.V., Amsterdam, The Netherlands) and sampled at 512 Hz after a low‐pass anti‐aliasing filter (0–104 Hz) which was applied by the acquisition hardware. Recordings were conducted in dedicated laboratories in the University of Dublin and St. James's Hospital, Dublin.

#### 2.3.3 MRI data

Magnetic resonance data were available for 37 ALS patients (Schuster, Elamin, Hardiman, & Bede, 2016). Structural T1‐weighted MRI data were acquired on a 3 T Philips Achieva system with a gradient strength of 80 mT/m and slew rate of 200 T/m/s using an eight‐channel receive‐only head coil. They were obtained using a three‐dimensional inversion recovery prepared spoiled gradient recalled echo sequence with field‐of‐view = 256 × 256 × 160 mm^3^, spatial resolution = 1 mm^3^ (Schuster et al., 2016; Schuster, Hardiman, & Bede, 2017). MRI scans were individually screened for the presence of vascular alterations on fluid‐attenuated inversion recovery (FLAIR) and diffusion‐weighted imaging (DWI) sequences and patients with co‐morbid vascular white matter lesions were not included (Bede, Iyer, Finegan, Omer, & Hardiman, 2017).

#### 2.3.4 Disease severity and neuropsychology data

The revised ALS functional rating scale (ALSFRS‐R) scores (Cedarbaum et al., 1999) from 61 patients, and contemporaneous scores from a standardised neuropsychological battery (Abrahams, Newton, Niven, Foley, & Bak, 2014; Pinto‐Grau et al., 2017) from 34 patients were obtained for clinico‐neurophysiological correlations. The scores from the neuropsychological battery were standardised into *z*‐scores, adjusting for age and education from a sample of 100 healthy controls.

### 2.4 Data analysis

#### 2.4.1 EEG data preprocessing

An automatic artefact rejection method (Dukic et al., 2017) based on statistical thresholding was used to discard data contaminated by noise (controls: mean 10%, range 2–19%; ALS: mean 11%, range 4–24%). After visual inspection, channels with higher levels of noise (controls: mean 2, range 0–7; ALS: mean 3.8, range 0–10) were removed and then interpolated from the rest of the electrodes using spherical spline interpolation (Perrin, Pernier, Bertrand, & Echallier, 1989). Data were band‐pass (1–97 Hz) and notch (50 Hz) filtered, and referenced to common average.

#### 2.4.2 EEG source localisation

EEG data were source reconstructed using the linearly constrained minimum variance beamformer (Van Veen, Van Drongelen, Yuchtman, & Suzuki, 1997) to obtain time‐varying signals originating from the brain. An atlas‐based approach was applied to estimate signals from 90 brain regions (see Supporting Information). The included regions of interest (ROIs) were from the automated anatomical labelling atlas (Tzourio‐Mazoyer et al., 2002), excluding the cerebellum and including the subcortical regions (see Figure S2, Supporting Information).

#### 2.4.3 Estimating spectral power

For each ROI, spectral power was calculated using the autospectrum:

$$S_{x}={\left|\text{FT}\left\{x\left(t\right)\right\}\right|}^{2}$$

where *x*(*t*) is a time‐domain signal corresponding to brain region and FT{∙} is a Fourier transformation. Spectral power was estimated in six frequency bands, as the sum of the auto‐spectrum values within each frequency band.

#### 2.4.4 Estimating functional connectivity

For each pair of ROIs, functional connectivity was calculated from two different perspectives to inform on different aspects of connectivity between brain regions.

An amplitude‐based measure, the ‘amplitude envelope correlation’ (AEC) (Brookes et al., 2011) measures the correlation between the power envelopes of two oscillatory time series. It reflects the simultaneous presence and co‐modulation of the intensity of the oscillatory activity in two regions. The phase synchrony of the oscillations in the two ROIs is not reflected in AEC. This amplitude‐based measure was chosen because of its capability to mirror the functional networks obtained in fMRI studies (Hipp & Siegel, 2015).

A phase‐based measure, the ‘imaginary coherence’ (iCoh) (Nolte et al., 2004), captures the extent to which two signals have a constant relative nonzero phase. This measure reflects the neuronal communication between the brain regions that contribute to synchronous neural oscillations, even though the intensity of the activities in the two ROIs may behave differently.

Estimating the functional connectivity in source‐space requires caution, since signals beamformed at spatially separate cortical locations are not necessarily independent (Schoffelen & Gross, 2009). This signal leakage can lead to spurious zero‐lagged connectivity between reconstructed signals (Palva et al., 2018). Hence, removing instantaneous relationships between pairs of projected signals would mitigate the problem, albeit at the expense of removing true instantaneous interactions between them. The implementation of the connectivity measures in this study corrects for this zero‐lag leakage.

#### 2.4.5 Amplitude envelope correlation

To mitigate the problem of spurious connectivity caused by source localisation methods, we performed time‐domain orthogonalisation of the reconstructed time series (Brookes, Woolrich, & Barnes, 2012) between each pair of ROIs before estimating the power envelopes, as follows:

$$p_{x}=\left|\mathrm{HT}\left\{x\left(t\right)\right\}\right|$$

$$p_{y}=\left|\mathrm{HT}\left\{y\left(t\right)\right\}\right|$$

where *x*(*t*) and *y*(*t*) are time‐domain signals representing two brain regions and filtered to a specific frequency band, and HT{∙} is a Hilbert transformation. Estimated power envelopes were then downsampled to 0.5 Hz. As a measure of association, an absolute value of Pearson's correlation was used on the entire log‐transformed power time series, as follows:

$$\mathrm{AEC}=\left|\text{corr}\left(\log\left(p_{x}\right),\log\left(p_{y}\right)\right)\right|$$

#### 2.4.6 Imaginary coherence

Unlike AEC, iCoh is not affected by the limitation of source localisation methods (Palva et al., 2018). It is defined as follows:

$$\text{iCoh}\left(f\right)=|Jm\left\{S_{\mathrm{xy}}\left(f\right)\right\}|/\sqrt{S_{x}\left(f\right)S_{y}\left(f\right)}$$

where $Jm\left\{S_{\mathrm{xy}}\right\}$ denotes the imaginary part of cross‐spectral density between the signal *x*(*t*) and *y*(*t*), whereas *S*_*x*_ and *S*_*y*_ are the auto‐spectral densities calculated for those signals. iCoh was estimated from 2 s long epochs.

These two measures were calculated for all possible pairs of estimated ROI signals, resulting in two symmetric 90 × 90 connectivity matrices for each subject. This was carried out for six separate frequency bands: δ (2–4 Hz), θ (5–7 Hz), α (8–13 Hz), β (14–30 Hz) and γ (γ_l_: 31–47 Hz, γ_h_: 53–97 Hz). Frequencies of 48–52 Hz were excluded from the analysis due to the potential power‐line noise. This resulted in 12 connectivity matrices per subject which are referred to as ‘point‐to‐point connectivity’. These matrices can be seen as weighted network matrices with elements representing link weights between network nodes. Additionally, each matrix was averaged using algebraic mean across ROIs to estimate the average connectivity of each brain region. This resulted in one value per ROI, representing mean node strength (average link weight), from each connectivity matrix.

#### 2.4.7 Connectivity modules

To extract and compare the connectivity modules/networks affected in ALS, we used non‐negative matrix factorisation (NMF) (Paatero & Tapper, 1994). This method factorises a given matrix *V*, such that *V*_*n* × *m*_ ≈ *W*_*n* × *k*_ ∙ *H*_*k* × *m*_, where matrices *W* (weight vectors) and *H* (basis vectors) are non‐negative and *n*, *k* and *m* denote the number of subjects, number of basis vectors and number of all possible brain connections, respectively. The non‐negativity constraint makes the NMF purely additive and therefore, suitable for the decomposition of complex connectivity patterns (*V*_*n* × *m*_) into *k* basis vectors (i.e., connectivity modules or networks) represented as a positive connectivity matrix (*H*_*k* × *m*_). Additionally, weight matrix (*W*_*n* × *k*_) informs us of the level of activation of each network per subject.

Factorisation of concatenated connectivity data from both healthy controls and ALS patients was used to reveal latent discriminant networks that are altered in ALS. To identify the networks that are affected in ALS, we applied NMF on all point‐to‐point connections that reached significant difference (Mann–Whitney *U* test, *p* < .05) between healthy controls and ALS patients. Factorisation was applied on a data matrix containing an equal number (*n* = 47) of healthy controls and ALS patients (selected at random) to avoid any bias towards one of the groups. The random sampling of ALS patients was repeated 250 times and the matrices *W* and *H* were averaged over the outcomes. At each run, to avoid the local minimum problem during the numerical solutions, multiple random starting values (*n* = 100) for *W* and *H* matrices were used. The number of modules to be extracted from the connectivity matrices was determined as the value that minimised the Bayesian information criterion (Schwarz, 1978).

#### 2.4.8 Correlates of EEG with MRI, neuropsychology and disease severity

We correlated the signal analysis findings with the structural MRI data, motor disease severity (ALSFRS‐R) and cognitive scores derived from a neuropsychological battery.

MRI studies have consistently shown motor and extra‐motor grey matter atrophy (Murphy, Henry, & Lomen‐Hoerth, 2007; Omer et al., 2017) and correlation between motor system pathology with ALSFRS‐R (Bede et al., 2013; Kassubek et al., 2018; Schmidt et al., 2014; Walhout et al., 2015). Consequently, we first sought to correlate the alterations in connectivity within motor and frontal networks (algebraic mean of the network connectivity matrix) with the cortical volume of those networks, where both motor and frontal networks are represented by several ROIs (see ‘Network definitions for correlation analysis’ section, Supporting Information).

Subsequently, the algebraic mean of the motor network connectivity matrix was correlated with the ALSFRS‐R scores. In addition, the scores from the neuropsychological battery were correlated with the alterations in frontoparietal and frontotemporal networks as captured by the weights corresponding to these networks from the NMF analysis. More specifically, we used the composite of summed executive function scores for the correlation with the alterations in frontoparietal network, and the composite of summed language function scores for the correlation with the alterations in frontotemporal network. The executive composite score included the following tests: Verbal fluency (Wechsler, 2014), Backward Digit span (Wechsler, 2014), the Delis‐Kaplan executive function system (D‐KEFS) Sorting Test (Delis, Kaplan, & Kramer, 2001), D‐KEFS Colour‐Word Interference Test (Delis et al., 2001), Reading the Mind in the Eyes (Baron‐Cohen, Wheelwright, Hill, Raste, & Plumb, 2001) and Conflicting Emotional Prosody from the Florida Affect Battery (Bowers, Blonder, & Heilman, 1991). The language composite score included: the PALPA (Auditory Lexical Decision, Visual Lexical Decision, Word Spelling, Word Reading, and Auditory Sentence‐Picture Matching and Written Sentence‐Picture Matching subtests) (Kay, Lesser, & Coltheart, 1992) and the Boston Naming test (Graves, Bezeau, Fogarty, & Blair, 2004).

In all cases, except in correlations with cognitive scores, the Spearman's partial correlation was used to test the presence of the hypotheses, and at the same time to correct for the age of patients. In the case of correlations with cognitive scores, the Spearman's correlation was used, since the cognitive scores were *z*‐scored, already accounting for age.

### 2.5 Statistical analysis

Statistical analysis of high‐dimensional measures suffers from high rates of false positive findings, which necessitates the use of advanced statistics to mitigate the problem. To determine the statistical significance of the observed differences in each of the three high‐dimensional measures (spectral power, AEC and iCoh), we used frequentist statistics together with an implementation of the empirical Bayesian inference (EBI) (Efron, 2007; Efron, Tibshirani, Storey, & Tusher, 2001) suited to neuroelectric signal analysis (Nasseroleslami, 2018). EBI provides major benefits, such as reliable estimation of FDR, calculation of statistical power and the posterior Bayesian probability, which are not afforded by alternative methods.

We used the area under the curve (AUC) of the receiver operating characteristics curve to make the between‐group comparisons (Zhou, McClish, & Obuchowski, 2011). To further infer statistical significance in a high‐dimensional space, EBI is applied on the test statistics (i.e., AUC), which exploits both the original (non‐null) observations and null‐permuted data to estimate the probability density function of the data and null, respectively. We then estimated the posterior probability (*P*_1_) and the statistical power (1‐β). The statistical analysis was performed separately for each of the three measures and for each frequency band in the case of point‐to‐point connectivity, while in the case of spectral power and average connectivity the analysis was done on the concatenated data across frequencies. False discovery rate (FDR) was set to 10%. This selection was based on careful inspection of curves that explain the relationship between FDR and power (or between Type I and Type II errors) as a function of thresholds (Nasseroleslami, 2018).

To assess the statistical significance and statistical power of the connectivity modules from the NMF analysis and the correlations between EEG connectivity and other measures, a null and non‐null bootstrapping resampling (*n* = 10,000) approach was applied (Nasseroleslami, 2018; Nasseroleslami et al., 2019). In the former case, the resampling was applied on the NMF weights, whereas in the latter case, it was applied on the data used in the correlation analysis. Here, to control for multiple comparisons, rejection of null hypothesis was additionally checked using adaptive FDR (aFDR) (Benjamini, Krieger, & Yekutieli, 2006) set to 5%.

In the analysis of ALS subgroups, two‐way ANOVA was applied on all three EEG measures (spectral power, AEC and iCoh) in two frequency bands that showed the most prominent changes in ALS patients compared to healthy controls. The analysis was performed on each measure and frequency band separately, with the *C9ORF72* status (presence/absence of the gene mutation) and the site of symptom onset (bulbar/spinal) chosen as independent variables. Data used were averaged from selected brain regions that had the highest discriminatory power between healthy controls and ALS patients in each measure and frequency band separately. Prior to the ANOVA analysis, data were transformed to standard normal distributions using the inverse normal transformation (Beasley, Erickson, & Allison, 2009; Efron, 2007).

The analyses were carried out in MATLAB (v2018a MathWorks Inc., Natick, MA) using the FieldTrip toolbox (Oostenveld, Fries, Maris, & Schoffelen, 2011), EBI toolbox (Nasseroleslami, 2018) and custom written scripts.

## 3 RESULTS

### 3.1 Spectral power revealed similar decrease across low‐ and mid‐frequency bands

**Figure 1:**
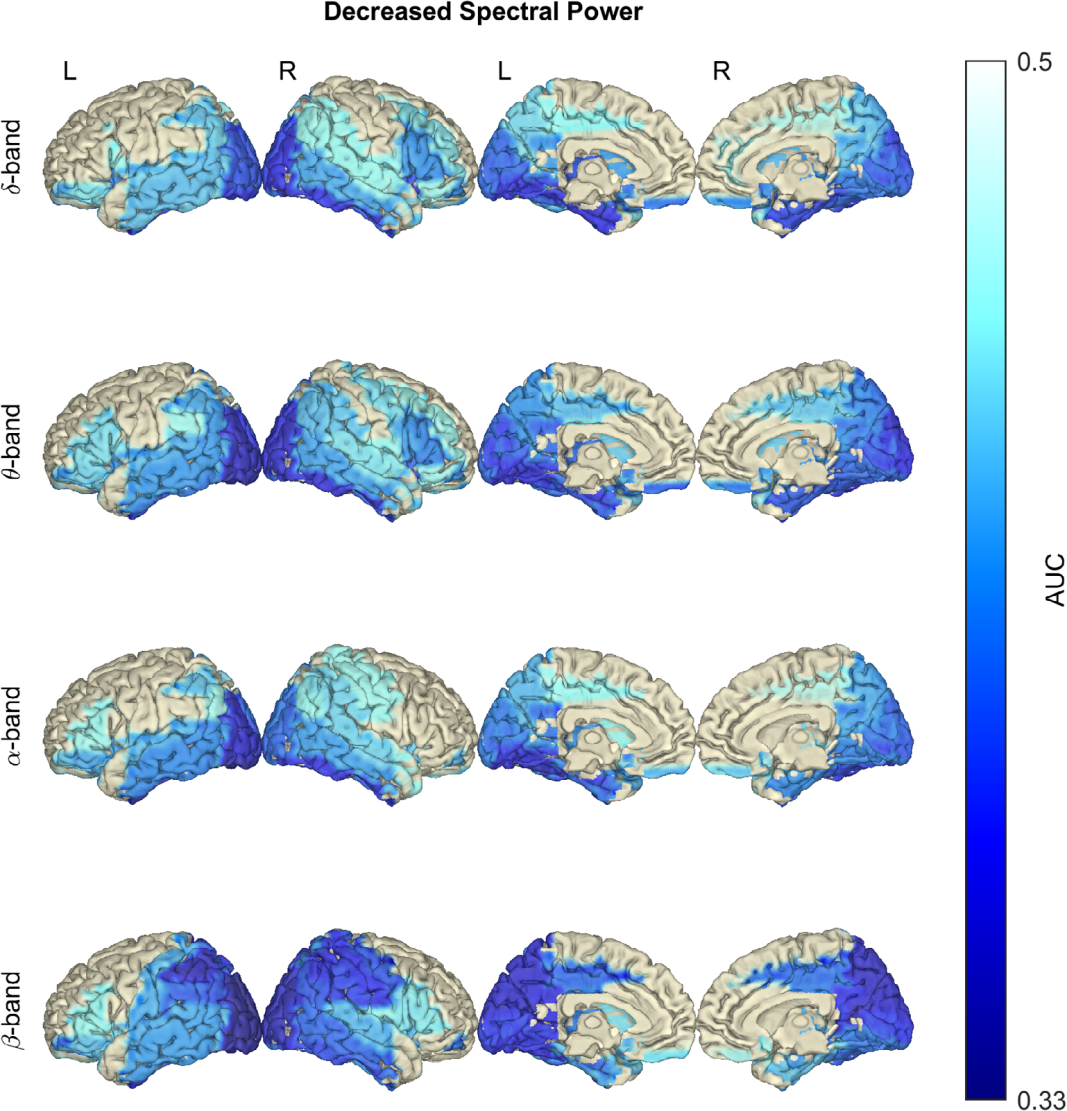
In amyotrophic lateral sclerosis (ALS), spectral power is significantly decreased between δ and β frequency bands. Notice the dominant decrease in the posterior and temporal regions. Statistical difference between healthy controls (*n* = 47) and ALS patients (*n* = 74) was assessed in the six defined frequency bands using empirical Bayesian inference (EBI). False discovery rate (FDR) was set to 10%, yielding an estimated statistical power of 1–β = .82 and posterior probability of *P*_1_ = .64 (across all frequency bands). AUC, area under the receiver operating characteristic curve. No changes were detected in the γ frequency bands; therefore, they are not shown. Frequency bands: δ (2–4 Hz), θ (5–7 Hz), α (8–13 Hz) and β (14–30 Hz) [Color figure can be viewed at http://wileyonlinelibrary.com]

Spectral power in ALS was significantly decreased and widespread from δ‐ to β‐band (Figure 1). The most notable changes were found in the occipital and temporal (from δ‐ to β‐band), lateral/orbitofrontal (δ‐ and θ‐band) and sensorimotor (β‐band) regions.

### 3.2 Average connectivity reflects frequency‐dependent changes in co‐modulation and synchrony

#### 3.2.1 Changes in the AEC revealed increased co‐modulation at δ, θ and γ_l_ bands

**Figure 2:**
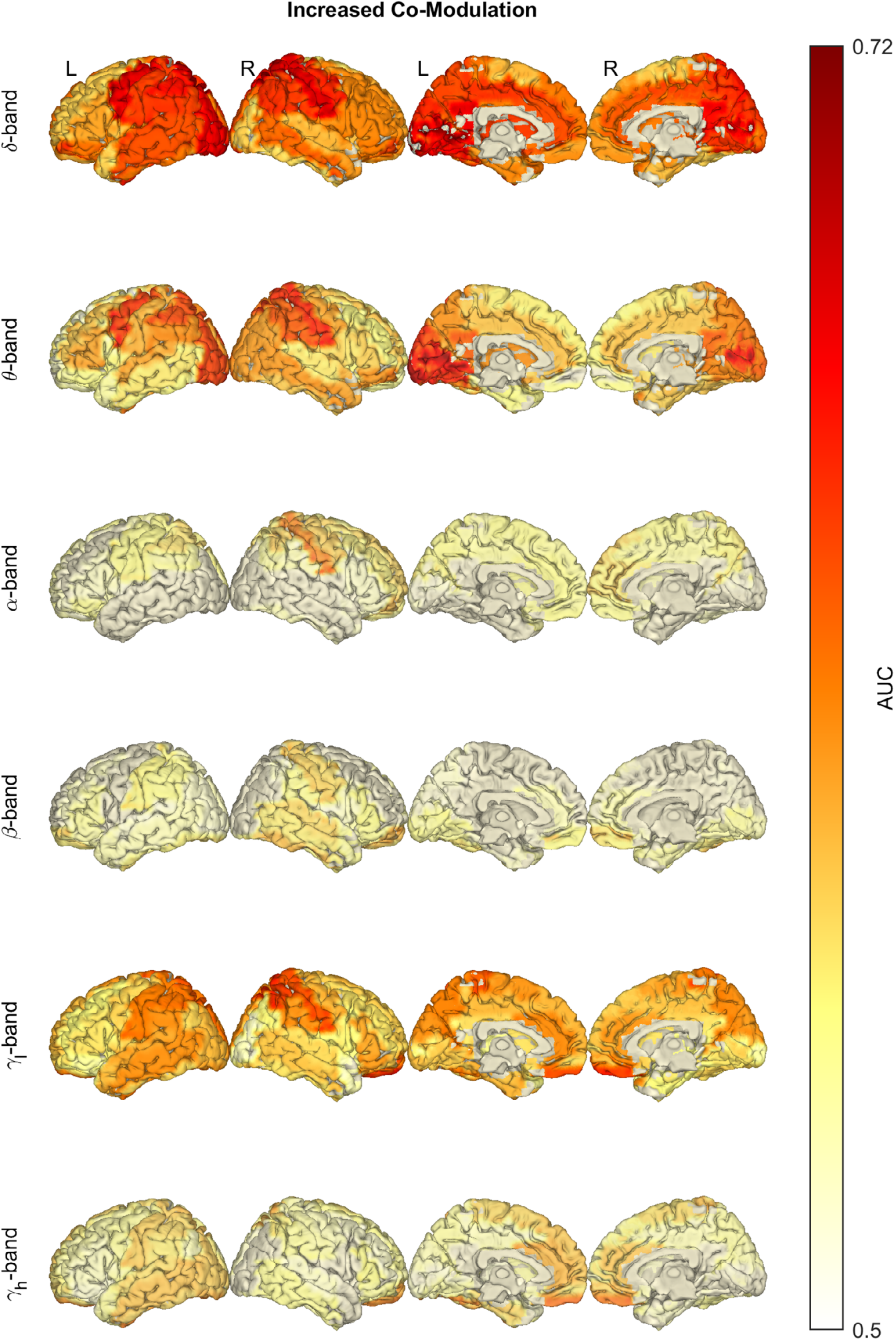
In amyotrophic lateral sclerosis (ALS), the average co‐modulation is significantly increased in the δ, θ and γ frequency bands. Notice the increase of amplitude envelope correlation (AEC) in the central and posterior regions (δ‐, θ‐ and γ_l_‐band) and frontal regions (δ‐ and γ_l_‐band). Statistical difference between healthy controls (*n* = 47) and ALS patients (*n* = 74) was assessed in the six defined frequency bands using empirical Bayesian inference (EBI). False discovery rate (FDR) was set to 10%, yielding an estimated statistical power of 1–β = .93 and posterior probability of *P*_1_ = .71 (across all frequency bands). AUC, area under the receiver operating characteristic curve. Frequency bands: δ (2–4 Hz), θ (5–7 Hz), α (8–13 Hz), β (14–30 Hz) and γ (γ_l_: 31–47 Hz, γ_h_: 53–97 Hz) [Color figure can be viewed at http://wileyonlinelibrary.com]

ALS patients showed a significant and widespread increase in AEC connectivity compared to healthy controls (Figure 2), with most notable changes in the central and posterior (δ‐, θ‐ and γ_l_‐band), and frontal (δ‐ and γ_l_‐band) regions.

#### 3.2.2 Changes in iCoh revealed decreased synchrony at δ and β bands

**Figure 3:**
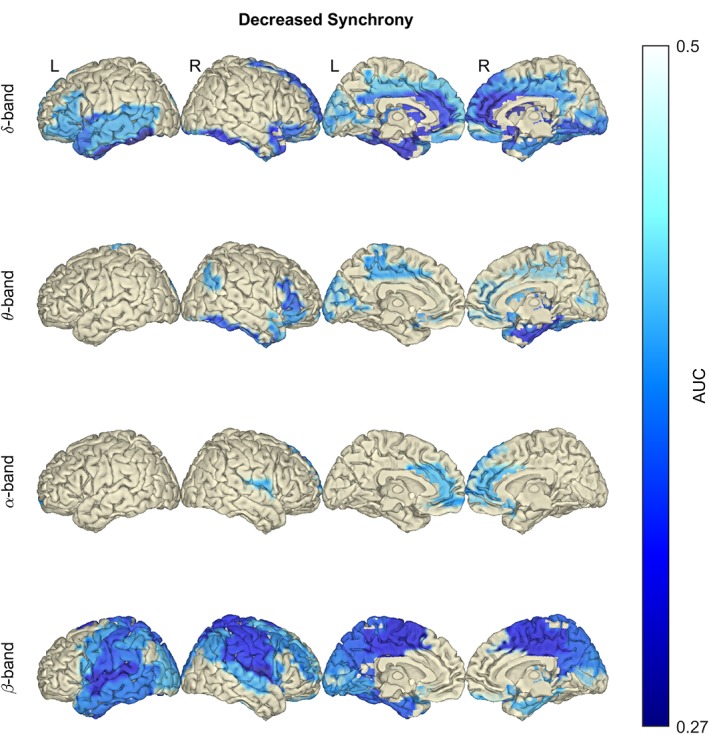
In amyotrophic lateral sclerosis (ALS), the average synchrony is significantly decreased in the δ and β frequency bands. Notice the decrease of imaginary coherence (iCoh) in temporal and frontal lobes (δ‐, θ‐ and α‐band), and in the sensorimotor cortex (β‐band). Statistical difference between healthy controls (*n* = 47) and ALS patients (*n* = 74) was assessed in the six defined frequency bands using empirical Bayesian inference (EBI). False discovery rate (FDR) was set to 10%, yielding an estimated statistical power of 1–β = .55 and posterior probability of *P*_1_ = .77 (across all frequency bands). AUC, area under the receiver operating characteristic curve. No changes were detected in the γ frequency bands; therefore, they are not shown. Frequency bands: δ (2–4 Hz), θ (5–7 Hz), α (8–13 Hz) and β (14–30 Hz) [Color figure can be viewed at http://wileyonlinelibrary.com]

ALS patients showed significant decrease in iCoh connectivity across multiple frequency bands compared with healthy controls (Figure 3). The changes were observed in temporal and frontal lobes from δ‐ to β‐band, while in β‐band, the decreased connectivity was additionally observed in the sensorimotor cortex. For an overview of results from the statistical analysis of spectral power, and average co‐modulation and synchrony, see Figure S3, Supporting Information.

### 3.3 Changes in point‐to‐point connectivity patterns are widespread

**Figure 4:**
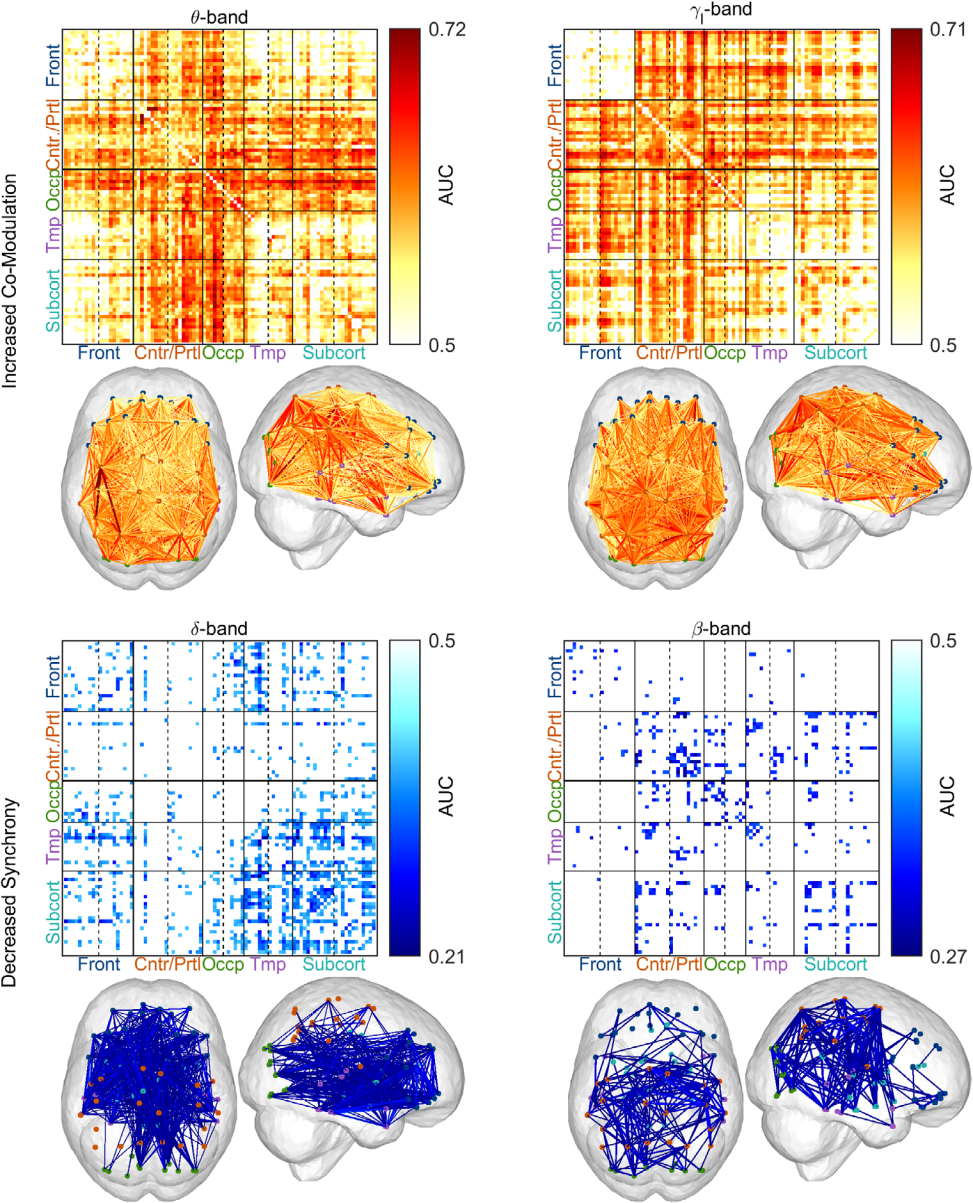
The increase of point‐to‐point co‐modulation and the decrease of point‐to‐point synchrony have a widespread pattern in amyotrophic lateral sclerosis (ALS) patients. Note that the widespread patterns of increased co‐modulation [amplitude envelope correlation (AEC)] are predominantly in the θ‐ and γ_l_‐bands, while synchrony [imaginary coherence (iCoh)] patterns were predominantly in the δ‐ and β‐bands. Statistical difference between healthy controls (*n* = 47) and ALS patients (*n* = 74) was assessed separately in the six defined frequency bands using empirical Bayesian inference (EBI). False discovery rate (FDR) was set to 10% (in each frequency band), yielding an estimated statistical power of 1–β = .96 and posterior probability of *P*_1_ = .56 in the θ‐band AEC and an estimated statistical power of 1–β = .89 and posterior probability of *P*_1_ = .7 in the γ_l_‐band AEC. For synchrony measures, the 10% FDR threshold yielded an estimated statistical power of 1–β = .39 and posterior probability of *P*_1_ = .8 in the δ‐band iCoh and an estimated statistical power of 1–β = .16 and posterior probability of *P*_1_ = .83 in the β‐band iCoh. AUC, area under the receiver operating characteristic curve. No changes were detected in the other frequency bands; therefore, they are not shown. The abbreviations ‘Front’, ‘Cntr/Prtl’, ‘Occp’, ‘Tmp’ and ‘Subcort’ stand for frontal, central/parietal, occipital, temporal and subcortical, respectively. For the order of ROIs used in the connectivity matrix, see Figure S2, Supporting Information. Frequency bands: δ (2–4 Hz), θ (5–7 Hz), β (14–30 Hz) and γ_l_ (31–47 Hz) [Color figure can be viewed at http://wileyonlinelibrary.com]

Significant widespread increase in the point‐to‐point co‐modulation of the neural activity was found in ALS patients compared with healthy controls in θ‐ and γ_l_‐band (Figure 4, upper). The θ‐band co‐modulation was observed within the regions encompassing the central, parietal and occipital lobe, as well as between these regions and the remainder of the brain; whereas the γ_l_‐band co‐modulation was present in the whole brain, but to a lesser extent within the frontal, between frontal and subcortical, and within and between temporal and subcortical regions.

Conversely, we detected significant decreases in the point‐to‐point synchrony in ALS patients compared with healthy controls in δ‐ and β‐band (Figure 4, lower). As it was the case for co‐modulation, the changes in synchrony were widespread—the δ‐band synchrony was decreased within the frontal regions, between the frontal and the occipital, temporal and subcortical regions, as well as between and within the temporal and subcortical regions; the β‐band synchrony was primarily decreased between and within the central and parietal regions and the rest of the brain (except the frontal region), and to a lesser extent within the occipital and subcortical regions.

### 3.4 The connectivity modules describe the (sub‐)networks affected in ALS

**Figure 5:**
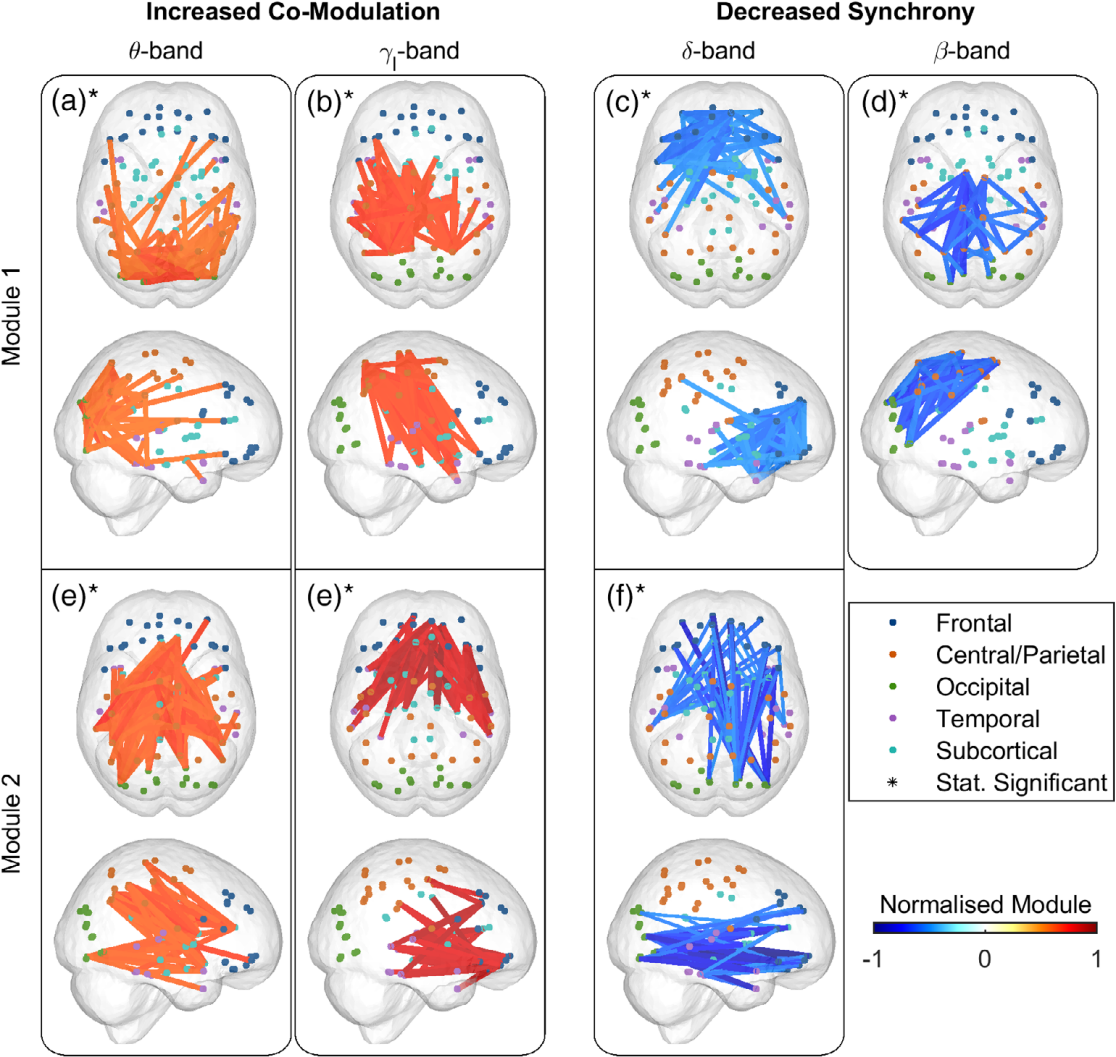
The connectivity modules reveal the (sub‐)network with frequency‐specific increase of co‐modulation and decrease of synchrony in amyotrophic lateral sclerosis (ALS). The factorised (sub‐)networks resemble the occipital network (a), motor loops of basal ganglia and/or thalamus (b), frontal network (c), sensorimotor network (d), frontoparietal network (e), frontotemporal network (f) and combined occipitofrontal and uncinate fasciculus (g). The connectivity modules from non‐negative matrix factorisation analysis of the affected co‐modulation or synchrony in ALS reveal the altered brain networks, while the changes in module's weights show the increase or decrease in the activity of these networks. Statistical analysis between ALS patients and healthy controls (*n*_c_ = 47 and *n*_p_ = 47) of the weights corresponding to the connectivity modules reached significance in all cases (marked with asterisk) as controlled by adaptive false discovery rate (aFDR) at *q* = 0.05. Frequency bands: δ (2–4 Hz), θ (5–7 Hz), β (14–30 Hz) and γ_l_ (31–47 Hz) [Color figure can be viewed at http://wileyonlinelibrary.com]

Considering the nonspecific widespread connectivity patterns identified in point‐to‐point analysis, we sought to further analyse the complex patterns into distinct networks. The Bayesian information criterion determined the preferred number of connectivity modules (M) to be one or two in each frequency band and measure. The factorised networks (i.e., the basis vectors extracted by NMF) resemble the occipital network (θ‐AEC M1; Figure 5a), motor loops of basal ganglia and/or thalamus (γ_l_‐AEC M1; Figure 5b), frontal network (δ‐iCoh M1; Figure 5c), sensorimotor network (β‐iCoh M1; Figure 5d), frontoparietal network (θ‐AEC M2; Figure 5e), frontotemporal network (γ_l_‐AEC M2; Figure 5f) and combined occipitofrontal and uncinate fasciculus (δ‐iCoh M2; Figure 5g).

Statistical analysis of the weights corresponding to the modules of AEC and iCoh showed the networks that were significantly different in the ALS patient group. Namely, weights corresponding to θ‐AEC M1 (*p* < .001; 1‐β_0.05_ = .99) and M2 (*p* < .001; 1‐β_0.05_ = 1) and γ_l_‐AEC M1 (*p* < .001; 1‐β_0.05_ = .97) and M2 (*p* = .001; 1‐β_0.05_ = .89) were significantly higher in ALS patients. Conversely, weights corresponding to δ‐iCoh M1 (*p* < .001; 1‐β_0.05_ = .9) and M2 (*p* = .014; 1‐β_0.05_ = .65) and β‐iCoh M1 (*p* < .001; 1‐β_0.05_ = .99) were significantly lower in ALS patients. In all cases, the statistical significance and statistical power of the connectivity modules were estimated using a bootstrapping method and controlled for false positives with an aFDR set to 5%. Figure 5 points to the increased or decreased activity of each network in ALS, where all the network connections in a module were pooled, normalised to the range between 0 and 1, and then each network was multiplied with the sign of the AUC value (centred around zero). The AUC value for each network corresponds to NMF weights of both groups.

### 3.5 EEG differences between ALS patients and controls do not discriminate between ALS subgroups

**Figure 6:**
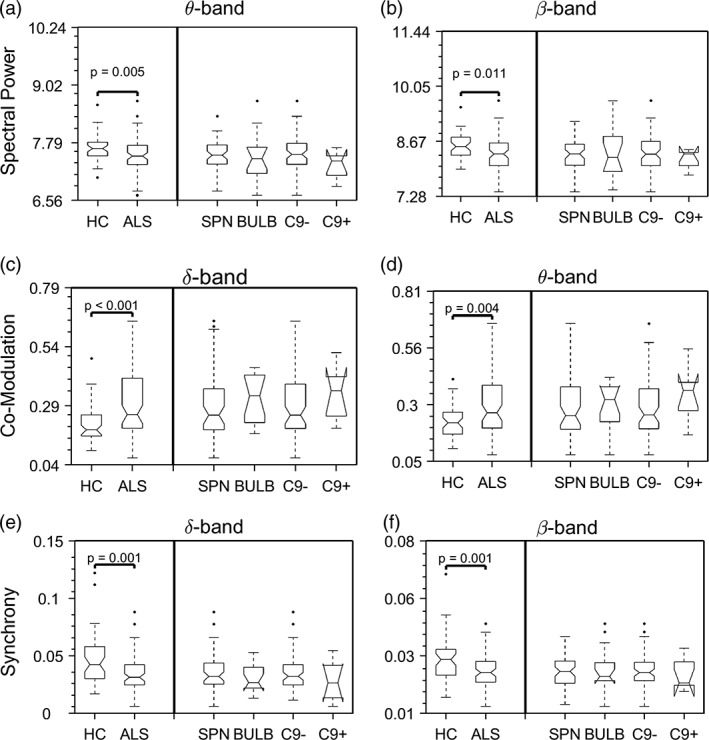
The observed electroencephalography (EEG) spectral power and connectivity changes are not different between amyotrophic lateral sclerosis (ALS) subgroups. The comparison shows the differences between healthy controls and ALS subgroups. Statistical difference between healthy controls and pooled ALS patients was assessed using Mann–Whitney *U* test, whereas statistical difference between patient subgroups was assessed using two‐way analysis of variance (ANOVA) in all three measures, each in two frequency bands with the most prominent changes (see Figures 1, 2, 3). None of the measures showed statistically significant difference among ALS subgroups. Spectral power data were log‐transformed for plotting purposes. The abbreviations ‘HC’, ‘SPN’, ‘BULB’, ‘C9‐’ and ‘C9+’ stand for healthy controls, spinal, bulbar, *C9ORF72*‐negative and *C9ORF72*‐postive, respectively. The number of ALS patients in each subgroup are *N* = 55, 15, 63 and 7, respectively. There are six *C9ORF72*‐postive patients in the spinal and one in the bulbar subgroup. Frequency bands: δ (2–4 Hz), θ (5–7 Hz) and β (14–30 Hz)

To assess the differences between ALS subgroups based on site of onset (spinal, bulbar) and the presence or absence of the pathologic hexanucleotide expansion in *C9ORF72*, we used EEG measures that discriminated between ALS patients and healthy controls (Figure 6). Brain regions that reached the highest AUC values between ALS patients and healthy controls in each of the following measures include: spectral power in θ‐band (L/R occipital gyri) and β‐band (L/R post‐central gyri and precuneus); co‐modulation in δ‐band (L/R post‐central and superior parietal gyri) and θ‐band (L/R calcarine fissure and lingual gyri); synchrony in δ‐band (L/R anterior cingulate gyri) and β‐band (L/R pre‐ and post‐central gyri).

Although these measures showed difference between ALS patients and healthy controls, they did not discriminate between subgroups based on site of onset (*p*_1_) or genomic status (*p*_2_) in any of the measures [i.e., spectral power in θ‐band (*p*_1_ = .471; *p*_2_ = .218) and β‐band (*p*_1_ = .484; *p*_2_ = .301); co‐modulation in δ‐band (*p*_1_ = .554; *p*_2_ = .445) and θ‐band (*p*_1_ = .708; *p*_2_ = .267); synchrony in δ‐band (*p*_1_ = .826; *p*_2_ = .35) and β‐band (*p*_1_ = .409; *p*_2_ = .717)].

### 3.6 The EEG measures of connectivity change reflect the neurodegeneration and functional impairment in both motor and cognitive domains

**Figure 7:**
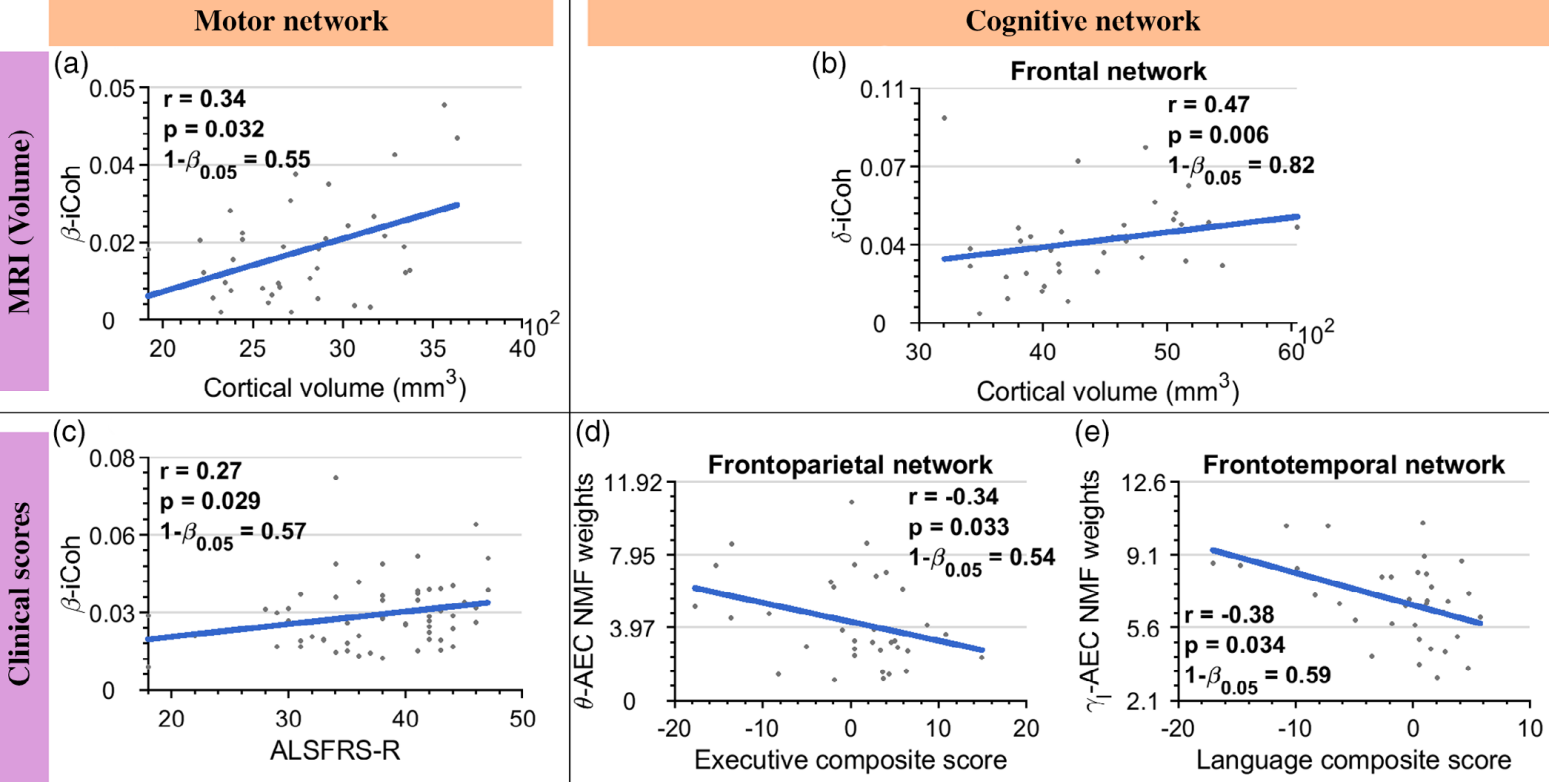
The changes in electroencephalography (EEG) connectivity correlate with the structural atrophy in MRI in the motor (a) and cognitive (b) networks, as well as measures of functional motor impairment [(c) amyotrophic lateral sclerosis Functional Rating Scale (ALSFRS‐R)], functional cognitive impairment [(d and e) standardised neuropsychological battery scores]. The values of *r* and *p* correspond to Spearman's partial correlation corrected for age (a–c) and Spearman's correlation (d and e), whereas 1‐β_0.05_ represents statistical power at 0.05. The number of ALS patients used in the analyses are *N* = 37, 37, 61, 34 and 34, respectively. The shown *p*‐values are adaptive false discovery rate (aFDR) corrected at *q* = 0.05. Frequency bands: δ (2–4 Hz), θ (5–7 Hz), β (14–30 Hz) and γ_l_ (31–47 Hz) [Color figure can be viewed at http://wileyonlinelibrary.com]

The changes in cortico‐cortical EEG connectivity were correlated with the changes captured by other modalities in both cognitive and motor domain; namely, the identified discriminant measures correlated with structural degeneration as captured by MRI, as well as the functional scores (ALSFRS‐R for motor function and neuropsychological battery scores for cognitive function). Figure 7 shows the significant correlations in each domain and for functional and structural measures.

For EEG–MRI (Figure 7a–b), these correlations were between the altered connectivity of motor and frontal networks with the grey matter volume of those networks. In the case of the motor network, the average motor network β‐band iCoh was correlated with the average cortical volume (Figure 7a), whereas in the case of the frontal network, the average δ‐band iCoh connectivity was correlated with average cortical volume (Figure 7b). We also found correlations between altered EEG connectivity and functional scores (Figure 7c–e). In the motor domain, the correlation was between the ALSFRS‐R scores with the average β‐band iCoh connectivity changes in the motor network (Figure 7c). In the cognitive domain, we found correlations between the neuropsychological battery scores and the alterations in the frontoparietal and frontotemporal networks (Figure 7d,e); Namely, between the composite of executive function scores and the θ‐band AEC NMF weights (frontoparietal network), as well as between the composite of language function scores and the γ_l_‐band AEC NMF weights (frontotemporal network).

All the correlations (Figure 7) were significant, as follows: between the changes in connectivity and grey matter volume in the motor network (*r* = .34; *p* = .032; 1‐β_0.05_ = .55) and frontal network (*r* = .47; *p* = .006; 1‐β_0.05_ = .82); changes in connectivity of the motor network and ALSFRS‐R (*r* = .27; *p* = .029; 1‐β_0.05_ = .57); changes in frontoparietal network with the executive function scores from the neuropsychological battery (*r* = −.34; *p* = .033; 1‐β_0.05_ = .54) and changes in frontotemporal network with the language scores from the neuropsychological battery (*r* = −.38; *p* = .034; 1‐β_0.05_ = .59).

## 4 DISCUSSION

This study demonstrates that neuroelectric signal analysis can capture and quantify important changes that occur in functional networks in ALS. Using spectral power and two conceptually different measures of connectivity that reflect co‐modulation (AEC) and synchrony (iCoh), we have demonstrated statistically robust neurophysiological evidence of a multisystem disruption of networks in ALS patients. These disruptions correlate with functional impairment as detected using ALSFRS‐R and neuropsychological assessment, as well as with structural changes captured by MRI.

### 4.1 Spectral power changes in ALS disease

The observed changes in spectral power are consistent with previously described θ‐ and α‐band power decrease above the sensorimotor network (Bizovičar, Dreo, Koritnik, & Zidar, 2014; Nasseroleslami et al., 2019). Other studies in ALS have similarly identified decreased post‐movement β‐band power above motor cortices (Proudfoot et al., 2017; Riva et al., 2012), which is considered a reflection of idling and/or an active inhibition of the motor network (Cassim et al., 2001).

Different frequency bands are mediated by complex neurochemistry and oscillations of frequencies 12–80 Hz are linked to pyramidal neurons, regulated by GABA_A_ inhibitory interneurons (Khanna & Carmena, 2015). Loss of GABAergic interneurons, together with pyramidal neurons, has been observed in both motor and nonmotor areas in ALS (Nihei, McKee, & Kowall, 1993); consequently, the decrease in the lower frequency spectral power can be attributed to structural degeneration of pyramidal cells and/or loss of interneurons that entrain them. These changes in spectral power observed beyond motor network (Bede et al., 2018; McMackin, Dukic, et al., 2019; Nasseroleslami et al., 2019) across multiple frequency bands, support the evolving recognition of significant involvement of nonmotor networks in ALS.

### 4.2 Correlating connectivity changes in networks affected by ALS with structural MRI and clinical scores

Our AEC connectivity findings are consistent with resting‐state fMRI findings of increased connectivity changes in the cingulate (Agosta et al., 2011; Douaud et al., 2011) and parietal cortices (Agosta et al., 2013), and prefrontal (Douaud et al., 2011), temporal and parahippocampal (Abrahams et al., 2004; Heimrath et al., 2014) regions in ALS patients. Factorised AEC networks from NMF analysis, showing increased connectivity in the θ‐ and γ_l_‐band, resemble the frontoparietal and frontotemporal networks, respectively.

The frontoparietal network is required for active maintenance of information relevant for successful performance in working memory (Ptak, 2012). Functional connectivity within this network (Blain‐Moraes et al., 2013; Iyer et al., 2015; Nasseroleslami et al., 2019), as well as the white matter volumes of association fibres within the frontal brain regions and cingulum are known to be affected in ALS, while the latter changes show correlation with memory impairments in ALS patients (Abrahams et al., 2005). In addition, degeneration of neurons in frontal and temporal regions (Bede et al., 2013) and associated tracts (Abrahams et al., 2005) is linked to language impairment (Neary et al., 1998) in ALS. We have identified significant correlations between our observed neurophysiological changes in the frontoparietal and frontotemporal network and composite scores of executive and language function, respectively. The observed negative correlation supports the role of such network dysfunction in cognitive impairment. These observations confirm the validity of neuroelectric signalling as a measure of clinically relevant network disruption in ALS.

Analysis of the iCoh networks using NMF showed that the most prominent changes are in the δ‐band frontal and β‐band sensorimotor networks, further demonstrating impaired connectivity in ALS patients. The identified positive correlation with structural MRI indicates that functional abnormalities we have detected are due to the loss of cortical atrophy in these regions. Taken together with a positive correlation between the average β‐band iCoh in the motor network and the ALSFRS‐R, these data support the use of advanced neurophysiological tools to characterise motor network decline.

### 4.3 Spectral EEG measures as a marker of ALS disease

Numerous studies in neurodegenerative diseases, particularly in dementias, have identified the potential of EEG resting‐state measures as biomarkers for differential diagnosis and clinical trial outcome measures (see the review by McMackin, Bede, Pender, Hardiman, & Nasseroleslami, 2019). By definition, ALS is a clinical diagnosis. The El Escorial criteria (Ludolph et al., 2015) often used in clinical practice allows for supportive evidence of both upper and lower motor neuron degeneration, the latter based on clinical neurophysiological studies. Diagnosis relies on physicians' expertise in marking specific set of symptoms corroborated by objective findings from laboratory, neurophysiological and neurological examination. Current clinical trial outcome measures similarly rely on semi‐quantitative tools, such as the ALSFRS‐R scale, a 48‐point clinical measure of motor, bulbar and respiratory decline. To date, there have been no reliable objective measures of cognitive/behavioural change suited for use as outcomes in clinical trials.

Quantitative EEG has the potential to capture upper motor system changes in ALS. Transcranial magnetic stimulation (TMS) studies have already demonstrated the utility of quantitative upper motor neuron biomarkers that distinguish ALS from mimic disorders (Vucic, Cheah, Yiannikas, & Kiernan, 2011). These TMS measures do not capture the broader, nonmotor degeneration established in ALS, such as that in cognitive networks. Conversely, EEG can capture both motor and nonmotor disruption. This is especially desirable from the clinical trial design perspective, where not only the severity of the disease, but specific (sub)phenotypes of disease that can be characterised by differential change in brain network architecture, are likely to be important in the evolution of a precision medicine approach towards treatments (McMackin, Muthuraman, et al., 2019). Our observed alterations in functional connectivity correlate with structural degeneration, and functional motor and cognitive measures. These changes confirm that neuroelectric signal analysis has a potential to be developed as a novel marker of ALS that reflects additional and previously uncharacterised dimensions of the disease, regardless of the site of onset. The high level of AUC values for the co‐modulation (0.72) and synchrony measures (0.27 or 0.73), show that these read‐outs can be used to develop quantitative biomarkers after further analysis of the sensitivity–specificity characteristics, establishing normative values, and further validation steps.

## 5 CONCLUSIONS

This study is the first to simultaneously interrogate power activity, co‐modulation and synchrony of brain networks in ALS to decipher the nature of change in network function caused by the disease using standard 128‐channel EEG recordings. In doing so, we have identified increased co‐modulation and decreased synchrony in both motor and nonmotor networks. Taken together, these data provide a compelling argument for the development of quantitative EEG, a noninvasive and inexpensive technology, as a robust data‐driven tool for measuring network disruption in ALS.

## Supporting information


**Appendix S1** Supporting Information

## Data Availability

The data that support the findings of this study are available from the corresponding author upon reasonable request and subject to the approvals by Data Protection Officer and Technology Transfer Office in Trinity College Dublin.
