# Supplementary material for: Patterned functional network disruption in amyotrophic lateral sclerosis
Source: Hum Brain Mapp. 2019 Jul 26;40(16):4827–42. doi: 10.1002/hbm.24740 (PMC6852475; doi:10.1002/hbm.24740)
Supplement: Supplementary file 1 — Appendix S1 Supporting Information [file HBM-40-4827-s001.docx]

# Supplementary material

## Supplementary methods

**EEG source localisation**: For source analysis, we pursued the following stages for head modelling, as well as projection of data to the source space in order to obtain time-varying signals, wherein each signal represents a brain region.

**Head models:** For each subject with an MRI scan, a realistically-shaped volume conduction model was built. A three-layer conduction model, accounting for the brain, skull and scalp, was constructed using the Boundary Element Models (BEM) method (Fuchs, Kastner, Wagner, Hawes, & Ebersole, 2002; Mosher, Leahy, & Lewis, 1999), implemented in the open source software OpenMEEG (Gramfort, Papadopoulo, Olivi, & Clerc, 2010). The BEMs were piece-wise approximated with 1000, 2000 and 3000 elements for scalp, skull and brain, respectively. Similarly, for controls and patients who did not undergo MRI, a realistically-shaped BEM based on the ICBM152 template (Fonov, Evans, McKinstry, Almli, & Collins, 2009) with the same characteristics was constructed, as template-based BEM and BEM based on individual MRI scans provide comparable localisation accuracy (Douw, Nieboer, Stam, Tewarie, & Hillebrand, 2018). For both groups, a 5 mm regular grid was generated in normalised space using the ICBM152 template and, separately for each subject, it was wrapped around the individual’s MRI data. Wrapping ensures that each potential source corresponds to the same location in the brain irrespective of individual anatomical differences. In addition, for each individual subject, a template with EEG electrode positions was realigned using the fiducial points, obtained manually from the individual’s MRI data. Aligned in the same coordinate system, these structures were used for the calculation of normalised leadfields. The use of normalised leadfield avoids the potential norm artefact of the leadfield, in which the norm of the leadfield changes with location (Jonmohamadi et al., 2014).

**Projection to source space:** EEG data were source-reconstructed using the linearly constrained minimum variance (LCMV) beamformer, a time-domain beamforming method (Van Veen, Van Drongelen, Yuchtman, & Suzuki, 1997).

Covariance matrices, needed for the reconstruction, were computed over a time window spanning the whole recording and using broadband data (1-97 Hz). In order to account for outliers in the data, the orthogonalised Gnanadesikan-Kettenring algorithm was used for the robust estimation of the covariance matrix (Maronna & Zamar, 2002). Covariance matrices were regularised using the Tikhonov method by $\mu I$ (Tikhonov & Arsenin, 1977), where the regularisation parameter, $\mu$, is set to 5% of the mean variance of all EEG channels and $I$ is an identity matrix. This was done to avoid reaching an unstable arbitrary solution, caused by reduction in dimensionality due to interpolation of noisy channels. Additionally, it increases temporal signal-to-noise ratio, albeit at the expense of increased spatial smoothness of the beamformed data.

At each grid point we sought to estimate a dipole in the optimal orientation. To achieve this, we estimated the orientation of maximum power of the dipole using the singular value decomposition (SVD) on the source-level covariance matrix (Van Veen et al., 1997). Beamformer weights, constructed for the source localisation, spatially filter the scalp recorded data and here were used to reconstruct time-series at each dipole location on the grid. One limitation of the LCMV beamformer is the overestimation of the sources in the centre of the head (Jonmohamadi et al., 2014; Van Veen et al., 1997). To compensate for this, we normalised the weights by their vector norm before reconstructing the time-series (Cheyne, Bostan, Gaetz, & Pang, 2007). Source localisation was done using the FieldTrip toolbox (Oostenveld, Fries, Maris, & Schoffelen, 2011).

Estimation of neural time-series at regions of interest (ROI): An atlas-based approach was used to evaluate source space data with respect to the anatomical brain regions (Hillebrand, Barnes, Bosboom, Berendse, & Stam, 2012; Tewarie et al., 2016). The cortex of each subject was parcelled according to the automated anatomical labelling (AAL) atlas (Tzourio-Mazoyer et al., 2002). This was done by using a 5 mm regular grid based on the ICBM152 template and labelling all the cortical sources according to the AAL atlas. In this study we used 90 ROI from AAL atlas, excluding the Cerebellum and including all the subcortical regions (olfactory cortex, insula, anterior/middle/posterior cingulate, hippocampus, parahippocampal gyrus, amygdala, caudate, putamen, globus pallidus and thalamus). For each ROI the centre of mass was calculated. To derive a single time-series for each ROI all the time-series within a ROI were weighted using a Gaussian weighting function with the half width at half maximum set to approximately 17 mm (Brookes et al., 2016; Tewarie et al., 2016), as following:

|  | $q_{1\times t}= \sum_{i} exp(\frac{-r^{2}(i)}{400})\cdot y_{1\times t}(i)$ |  |
| --- | --- | --- |

Where $i$ represents a count of all dipoles within a ROI, $r$ represents a distance in millimetres of each dipole from the centre of mass of the given ROI and $t$ represent the length of the reconstructed signal. This means that signals that are 17 mm far from the centre of the ROI will be attenuated by a factor of 0.5. However, the direction of each estimated dipole is not necessarily the same as the other dipoles in the ROI. In such situations, simple averaging of neighbouring dipoles’ time-series would lead to cancellations and incorrect estimation of the effective activity of the ROI. Therefore, before deriving a single time-series of each ROI, we estimated the direction along the maximum power for each region by performing singular value decomposition on the orientations of dipoles within each ROI. Dipoles with the opposite direction (>90 degrees) to the estimated ROI’s maximal activity vector were sign-flipped. After completing these steps, we obtained 90 broadband time-series, each representing one ROI from the AAL atlas. This pipeline was applied to each subject individually.

**Network definitions for correlation analysis:** Two different anatomical atlases were used in the correlation analysis: AAL atlas for the connectivity and Destrieux atlas (Destrieux, Fischl, Dale, & Halgren, 2010) for the structural MRI analysis.

The ROI used to represent the motor network in the connectivity analysis are: L/R supplementary motor area, L/R paracentral lobule, L/R precentral gyrus and L/R rolandic operculum; whereas for the MRI analysis are: L/R paracentral gyrus and sulcus, L/R subcentral gyrus and sulcus, L/R precentral gyrus, L/R central sulcus, L/R precentral inferior sulcus and L/R precentral superior sulcus.

Similarly, ROI used to represent the frontal network in both connectivity and MRI analyses are the anterior cingulate gyrus and all the frontal regions defined by AAL and Destrieux atlases, resepctively. Namely, in the former case the included ROI are: L/R anterior cingulate, L/R rectus, L/R frontal superior orbital, L/R frontal medial orbital, L/R frontal middle orbital, L/R frontal inferior orbital, L/R frontal superior, L/R frontal middle, L/R frontal inferior opercular, L/R frontal inferior triangular and L/R frontal superior medial gyri. In the latter case the included ROI are: L/R anterior cingulate gyrus and sulcus, L/R frontomarginal gyrus and sulcus, L/R transverse frontopolar gyrus and sulcus, L/R frontal inferior opercular gyrus, L/R frontal inferior orbital gyrus, L/R frontal inferior triangular gyrus, L/R frontal middle gyrus, L/R frontal superior gyrus, L/R frontal inferior sulcus, L/R frontal middle sulcus and L/R frontal superior sulcus.

## Supplementary figures


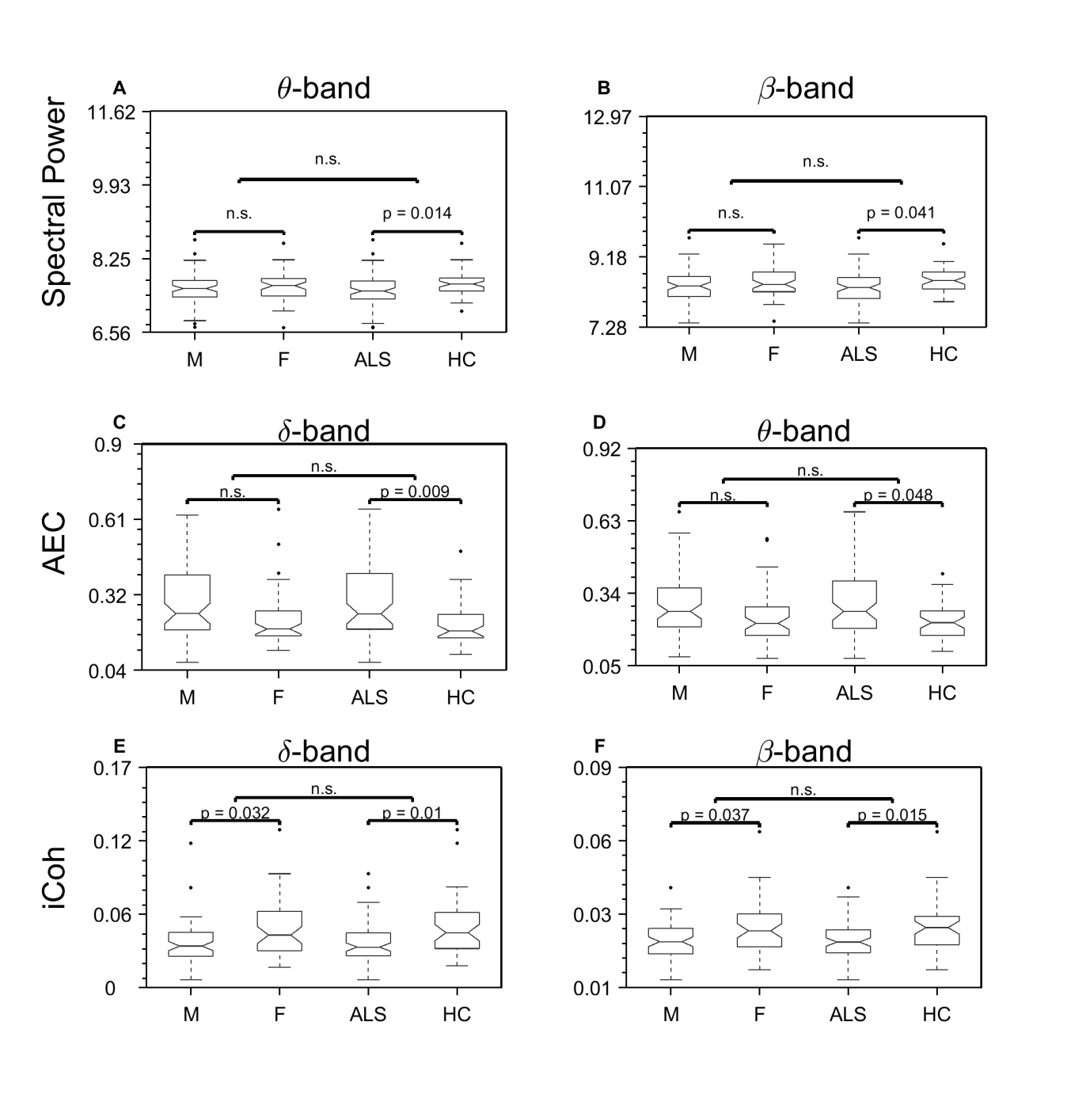


Supplementary Figure 1. The effects of gender and ALS disease in all three EEG measures in two frequency bands with the most prominent changes using 2-way ANOVA. Note that the interaction effects are not significant (n.s.) in all cases, eliminating the possibility of the gender-effect on the main findings. The statistical analysis, similar to the analysis of the ALS sub-groups, had two independent variables: gender (M/F) and group (HC/ALS). Prior to the analysis, data were transformed to standard normal distributions using the inverse normal transformation (Beasley, Erickson, & Allison, 2009; Efron, 2007). Spectral power data were log-transformed for plotting purposes. The abbreviations ‘M’, ‘F’, ‘HC’ stand for Male, Female and Healthy controls, respectively.


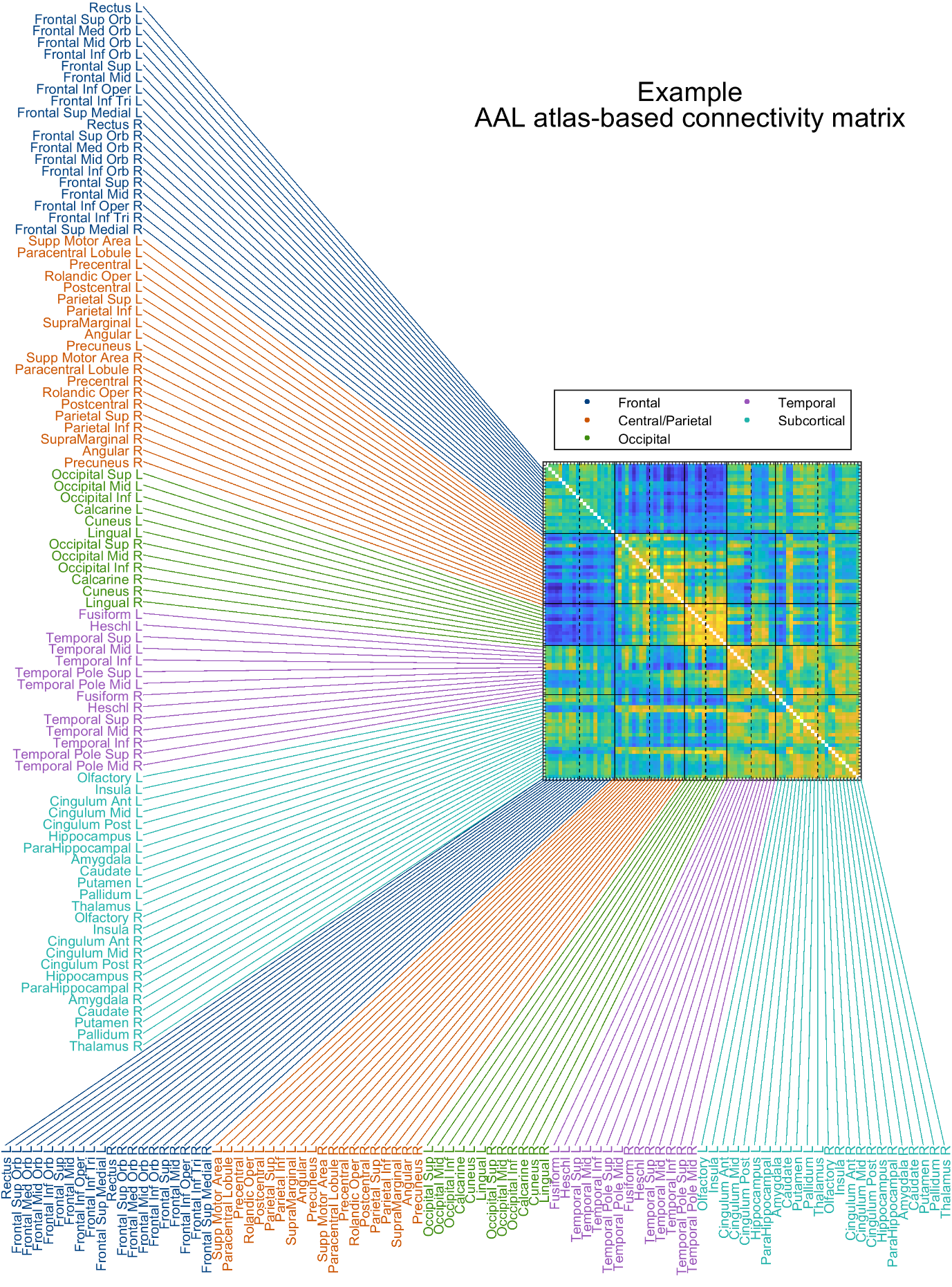


Supplementary Figure 2. An Example of an AAL atlas-based connectivity matrix. From the Automated anatomical labelling (AAL) atlas 90 brain regions (excluding those in cerebellum) were grouped and colour-coded in the following order: frontal (blue), central and parietal (orange), occipital (green), temporal (purple) and subcortical (cyan) regions. Each group has regions from the left (L) hemisphere first and then from the right (R). The same connectivity matrix organisation was used in co-modulation and synchrony figures. The figure corresponds to co-modulation in α frequency band in heathy controls.


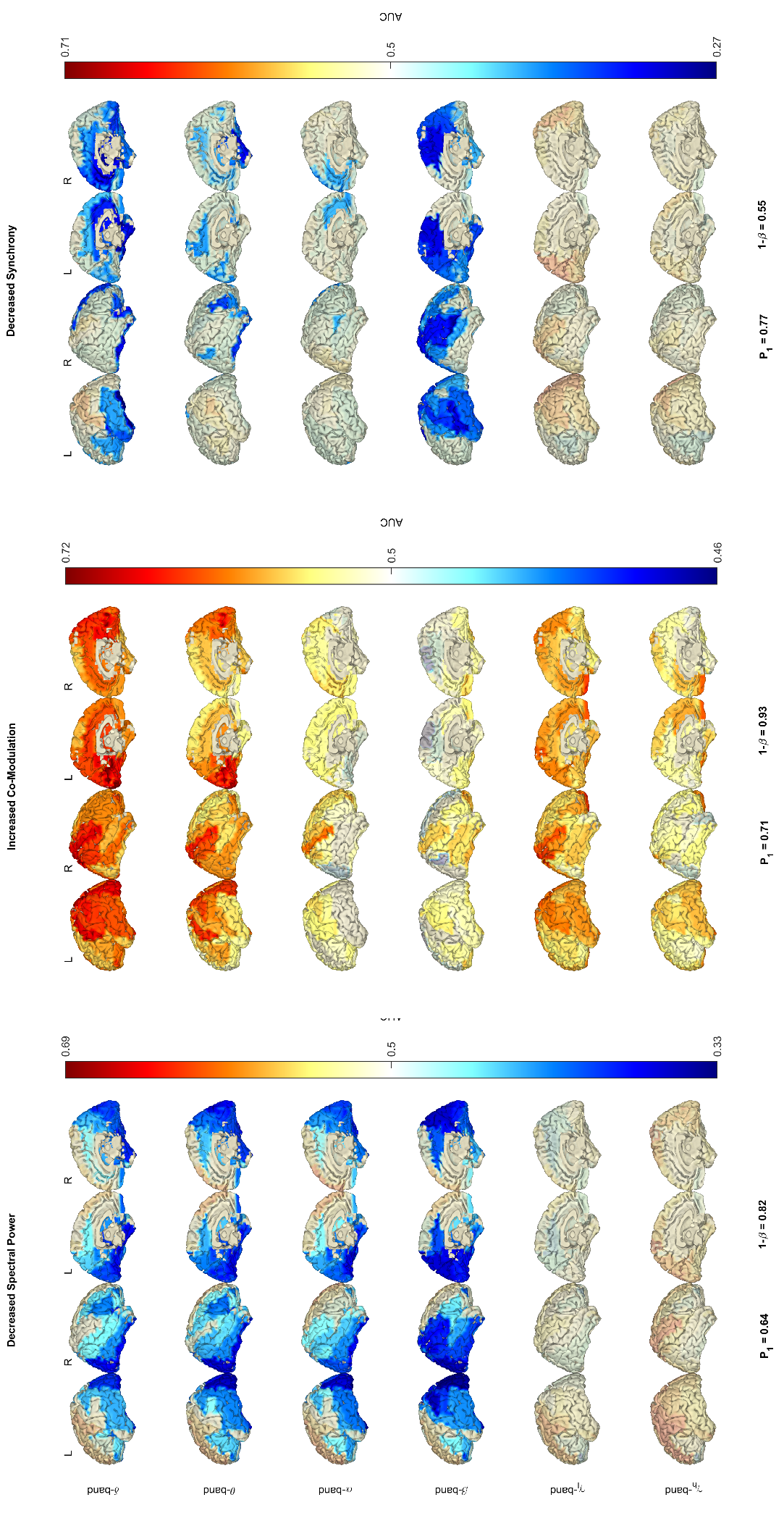


Supplementary Figure 3. A side-by-side overview of the results from the statistical analysis of spectral power and average brain connectivity. For each measure, statistical difference between healthy controls (n = 47) and ALS patients (n = 74) was assessed across six frequency bands using Empirical Bayesian Inference with false discovery rate set to 10%. Spectral power was decreased (δ-β), co-modulation increased (mostly δ, θ and γl) and synchrony was decreased (δ-β) significantly in ALS patients. Nonsignificant findings are presented with lower opacity. AUC: area under the receiver operating characteristic curve. Frequency bands: δ (2-4 Hz), θ (5-7 Hz), α (8-13 Hz), β (14-30 Hz) and γ (γl: 31-47 Hz, γh: 53-97 Hz).

# Supplementary material references

Beasley, T. M., Erickson, S., & Allison, D. B. (2009). Rank-Based Inverse Normal Transformations are Increasingly Used, But are They Merited? *Behavior Genetics*, 39, 580–95. https://doi.org/10.1007/s10519-009-9281-0

Brookes, M. J., Tewarie, P. K., Hunt, B. A. E., Robson, S. E., Gascoyne, L. E., Liddle, E. B., … Morris, P. G. (2016). A multi-layer network approach to MEG connectivity analysis. *NeuroImage*, 132, 425–38. https://doi.org/10.1016/J.NEUROIMAGE.2016.02.045

Cheyne, D., Bostan, A. C., Gaetz, W., & Pang, E. W. (2007). Event-related beamforming: a robust method for presurgical functional mapping using MEG. *Clinical Neurophysiology : Official Journal of the International Federation of Clinical Neurophysiology*, 118, 1691–704. https://doi.org/10.1016/j.clinph.2007.05.064

Destrieux, C., Fischl, B., Dale, A., & Halgren, E. (2010). Automatic parcellation of human cortical gyri and sulci using standard anatomical nomenclature. *NeuroImage*, 53, 1–15. https://doi.org/10.1016/J.NEUROIMAGE.2010.06.010

Douw, L., Nieboer, D., Stam, C. J., Tewarie, P., & Hillebrand, A. (2018). Consistency of magnetoencephalographic functional connectivity and network reconstruction using a template versus native MRI for co-registration. *Human Brain Mapping*, 39, 104–119. https://doi.org/10.1002/hbm.23827

Efron, B. (2007). Size, power and false discovery rates. *The Annals of Statistics*, 35, 1351–77. https://doi.org/10.1214/009053606000001460

Fonov, V., Evans, A., McKinstry, R., Almli, C., & Collins, D. (2009). Unbiased nonlinear average age-appropriate brain templates from birth to adulthood. *NeuroImage*, 47, S102. https://doi.org/10.1016/S1053-8119(09)70884-5

Fuchs, M., Kastner, J., Wagner, M., Hawes, S., & Ebersole, J. S. (2002). A standardized boundary element method volume conductor model. *Clinical Neurophysiology : Official Journal of the International Federation of Clinical Neurophysiology*, 113, 702–12. https://doi.org/10.1016/S1388-2457(02)00030-5

Gramfort, A., Papadopoulo, T., Olivi, E., & Clerc, M. (2010). OpenMEEG: opensource software for quasistatic bioelectromagnetics. *BioMedical Engineering OnLine*, 9, 45. https://doi.org/10.1186/1475-925X-9-45

Hillebrand, A., Barnes, G. R., Bosboom, J. L., Berendse, H. W., & Stam, C. J. (2012). Frequency-dependent functional connectivity within resting-state networks: An atlas-based MEG beamformer solution. *NeuroImage*, 59, 3909–21. https://doi.org/10.1016/J.NEUROIMAGE.2011.11.005

Jonmohamadi, Y., Poudel, G., Innes, C., Weiss, D., Krueger, R., & Jones, R. (2014). Comparison of beamformers for EEG source signal reconstruction. *Biomedical Signal Processing and Control*, 14, 175–88. https://doi.org/10.1016/j.bspc.2014.07.014

Maronna, R. A., & Zamar, R. H. (2002). Robust Estimates of Location and Dispersion for High-Dimensional Datasets. *Technometrics*, 44, 307–17. https://doi.org/10.1198/004017002188618509

Mosher, J. C., Leahy, R. M., & Lewis, P. S. (1999). EEG and MEG: forward solutions for inverse methods. *IEEE Transactions on Biomedical Engineering*, 46, 245–59. https://doi.org/10.1109/10.748978

Oostenveld, R., Fries, P., Maris, E., & Schoffelen, J.-M. (2011). FieldTrip: Open source software for advanced analysis of MEG, EEG, and invasive electrophysiological data. *Computational Intelligence and Neuroscience*, 2011, 156869. https://doi.org/10.1155/2011/156869

Tewarie, P., Bright, M. G., Hillebrand, A., Robson, S. E., Gascoyne, L. E., Morris, P. G., … Brookes, M. J. (2016). Predicting haemodynamic networks using electrophysiology: The role of non-linear and cross-frequency interactions. *NeuroImage*, 130, 273–92. https://doi.org/10.1016/J.NEUROIMAGE.2016.01.053

Tikhonov, A. N., & Arsenin, V. Y. (1977). *Solutions of Ill-Posed Problems*. New York: Halsted Pres.

Tzourio-Mazoyer, N., Landeau, B., Papathanassiou, D., Crivello, F., Etard, O., Delcroix, N., … Joliot, M. (2002). Automated Anatomical Labeling of Activations in SPM Using a Macroscopic Anatomical Parcellation of the MNI MRI Single-Subject Brain. *NeuroImage*, 15, 273–89. https://doi.org/10.1006/NIMG.2001.0978

Van Veen, B. D., Van Drongelen, W., Yuchtman, M., & Suzuki, A. (1997). Localization of brain electrical activity via linearly constrained minimum variance spatial filtering. *IEEE Transactions on Biomedical Engineering*, 44, 867–80. https://doi.org/10.1109/10.623056
